# Amelioration of ulcerative colitis *via* inflammatory regulation by macrophage-biomimetic nanomedicine

**DOI:** 10.7150/thno.48448

**Published:** 2020-08-08

**Authors:** Tianlei Sun, Cheryl H. T. Kwong, Cheng Gao, Jianwen Wei, Ludan Yue, Jianxiang Zhang, Richard Dequan Ye, Ruibing Wang

**Affiliations:** 1 State Key Laboratory of Quality Research in Chinese Medicine, Institute of Chinese Medical Sciences, University of Macau, Taipa, Macau 999078, China.; 2 School of Life and Health Sciences, The Chinese University of Hong Kong, Shenzhen, Guangdong 518172, China.; 3 Department of Pharmaceutics, College of Pharmacy, Third Military Medical University (Amy Medical University), Chongqing 400038, China.

**Keywords:** cyclodextrin, ROS-responsive, macrophage polarization, ulcerative colitis, biomimetic materials

## Abstract

Ulcerative colitis (UC) is featured with relapsing inflammation in the colon, where macrophages are recruited and polarized locally into M1 type to drive further inflammation. Pharmacotherapy of UC has exhibited limited efficacy, mostly due to the poor specificity.

**Methods:** A macrophage-biomimetic nanomedicine was developed for targeted treatment of UC, which was derived from reactive oxygen species (ROS)-sensitive β-cyclodextrin, loaded with rosiglitazone, and coated with macrophage membrane. The ability of the nanomedicine in regulating macrophage polarization was examined at cellular level, and the macrophage-tropism driven targeted delivery into the inflammatory colon was investigated by *ex vivo* bio-imaging distribution assay. Furthermore, the nanomedicine's therapeutic efficacy was systemically examined in dextran sulfate sodium (DSS)-induced colitis model in mice.

**Results:** The nanomedicine effectively polarized macrophages to M2 and protected epithelial cells from oxidative stress *in vitro*. In addition, macrophage-membrane led the nanomedicine to the inflammatory colon with a high targeting efficiency. In response to the elevated ROS in the inflammatory tissue, the nanomedicine released rosiglitazone specifically and regulated macrophage polarization *in vivo*. Macrophage membrane also assisted inflammation suppression by sequestering proinflammatory cytokines. Working in such a synergy, the nanomedicine exhibited significant therapeutic effects against UC in mice.

**Conclusions:** This macrophage-biomimetic nanomedicine leverages the inflammatory tropism and inflammatory cytokine sequestration effects of macrophage membrane for targeted delivery and local inflammation suppression, the ROS-responsiveness of β-cyclodextrin-based matrix for specific payload release, and the macrophage-polarizing effect of rosiglitazone for inflammatory regulation, thereby exhibiting considerable therapeutic efficacy against UC in mice. This study offers important new insights on the design and development of biomimetic nanomaterials for inflammation regulations.

## Introduction

Ulcerative colitis (UC) is a non-curable inflammatory colon disease, afflicting thousands of people worldwide. With the modern lifestyle and environmental exposure, the UC occurrence rate has rapidly increased, especially in the developed countries such as Europe and North America 1. The symptoms of UC, including diarrhea, weight loss, abdominal pain and rectal bleeding, significantly compromise the life quality of patients 2. As the etiology is not clearly elucidated, the clinical treatments by pharmacotherapy nowadays are not very satisfactory, reflected by a high relapse rate and elevated risk of carcinogenesis 3, 4, mainly due to the poor specificity and inadequate suppression of inflammation by majority of the anti-inflammatory drugs. Recently, many novel nanomedicines exhibited improved targeting efficiency and therapeutic effects in the treatment of UC 5, 6. Among a plethora of bionanomaterials, as was systemically reviewed by Liu et al., cyclodextrin-based nanoassemblies have exhibited significant potential in controlled drug delivery, attributed to their high biocompatibility and tunable functionality allowing responsiveness towards different stimuli 7. For instance, by taking advantages of the elevated ROS level in ulcerative colon 8, the ROS-responsive nanoparticles (NPs) based on β-cyclodextrin derivatives firstly developed by Zhang and Li et al exhibited an excellent safety profile, improved drug accumulation in the inflammatory colon, and enhanced the therapeutic efficacy against UC 9. With the same ROS-responsive nanomedicine platform, pro-resolving peptide Ac2-26 was released in the high ROS environment specifically in the UC colon, driving inflammatory resolution effectively 10. In spite of these significant progresses, many challenges still exist with these novel nanomedicines, such as low accumulation in the colon tissues, fast clearance, and little distinction in actions on different cell types.

Very recently, biomimetic drug delivery systems, particularly cell membrane coated NPs, have rapidly emerged and demonstrated improved treatment efficacy against cancer and inflammatory diseases 11-13, attributed to their highly biocompatible “self” nature and specific accumulation in targeted tissues and organs 14. Indeed, in addition to the high biocompatibility, different cell membranes coating equipped NPs with different functions. For instance, poly(lactic-co-glycolic acid) (PLGA) NPs coated with red blood cell (RBC) membrane developed by Zhang et al not only prolonged the systemic circulation of the NPs, but also absorbed pore-forming toxins in bacterial infection, thus protected RBC and prevented hemolysis 15, 16. Gu and coworkers developed biomimetic nanomedince where the hybrid membranes from RBC and tumor cells functioned as tumor-antigen carriers and enhanced immunotherapy in combination with anti-programmed death ligand 1 (PD-L1) 17. Of a particular relevance, macrophage membrane coated NPs not only accumulated in the inflammatory sites specifically due to their inflammation-homing effects 18, 19, but also absorbed lipopolysaccharide (LPS) and inflammatory cytokines to regulate and suppress local inflammation in the management of sepsis 20, and for the treatment of atherosclerosis 19. Therefore, macrophage-biomimetic nanomedicine may exhibit significant potential in targeting and managing a variety of inflammatory diseases including UC. However, most of the NPs in these studies usually do not release payload in a specific manner, which may result in off-target toxicity.

In addition, increased M1 and decreased M2 macrophages are commonly observed in the inflammatory colon tissues of UC patients, and the disequilibrium between M1 and M2 correlates with the progression of UC 21. Therefore, administration of *in-vitro* derived M2 macrophages into mice significantly reduced the colon inflammation 22. Under the same principle, effectively regulating macrophage polarization *in vivo* may become a potential therapeutic strategy for treating UC 6. Rosiglitazone, an agonist of peroxisome proliferator activated receptor γ (PPARγ) 23, was reported to induce M2 polarization of macrophages 24, 25, which contributed to its therapeutic effects against UC 26, 27.

**Scheme 1:**
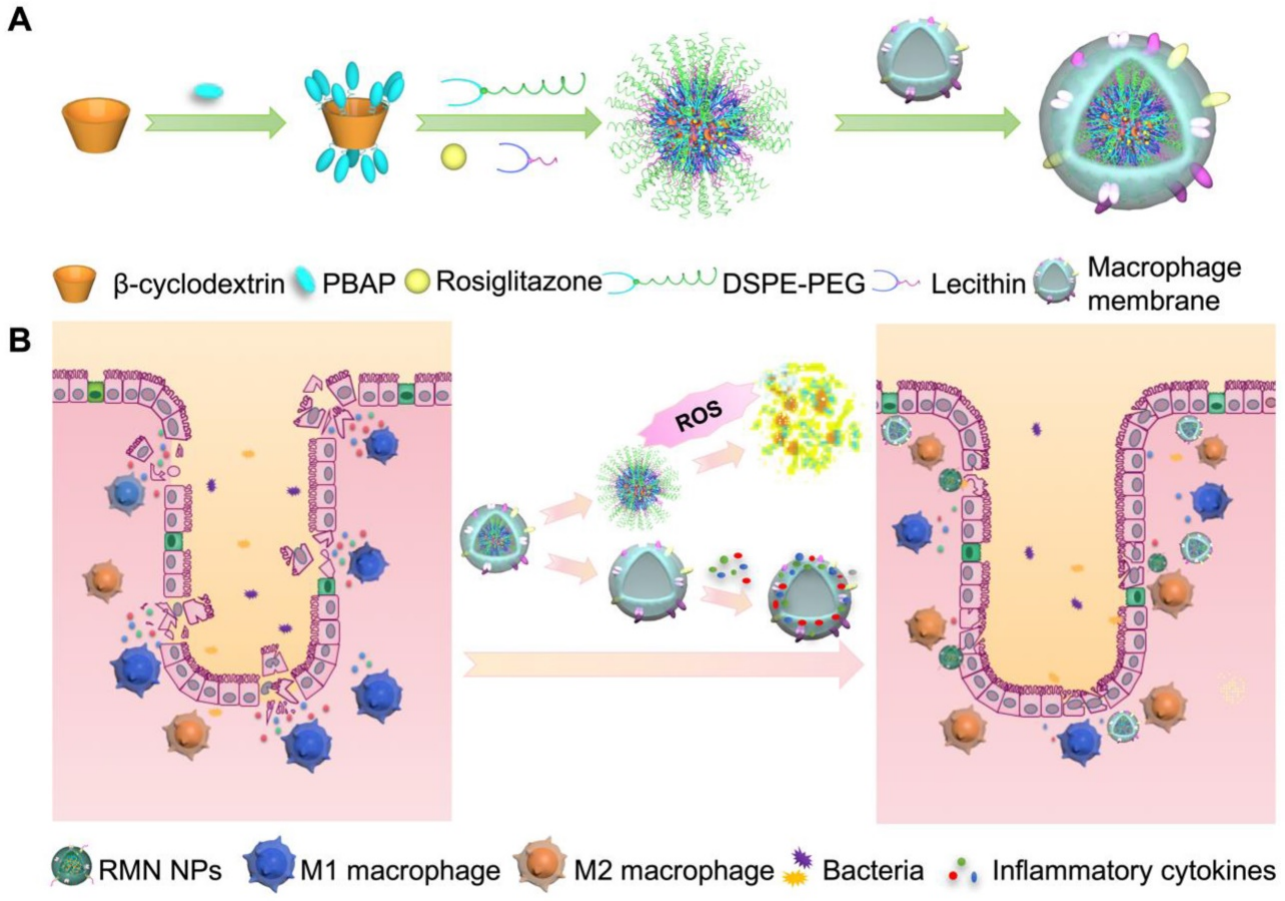
Fabrication process of RMN NPs (A), and targeted therapy of UC by RMN NPs (B).

In light of the inflammatory tropism and sequestration of inflammatory cytokines and chemokines by macrophage membrane 19, 20, the elevated ROS level in the inflammatory site of UC 8 and the potential inflammatory regulation by M2 macrophage 22, herein we developed a rosiglitazone (RLZ)-loaded, macrophage membrane coated, ROS-responsive nanomedicine (RMN NPs) based on ROS-sensitive β-cyclodextrin (Ox-CD), for targeted therapy of UC *via* synergistic regulation of inflammation (Scheme 1) upon intravenous administration. Macrophage membrane shells assist the NPs in targeting the inflammatory colon and absorb inflammatory mediators to suppress the inflammation. In response to the significantly high ROS level in the inflammatory site, NPs based on Ox-CD release the payload, RLZ, which subsequently polarizes macrophages to M2, thereby further regulating the inflammatory microenvironment. Working in such a synergy, the inflammation in the colon is dramatically alleviated *in vivo*.

## Methods

### Materials and Equipment

β-Cyclodextrin (β-CD), 4-phenylboronic acid pinacol ester (PBAP), 4-dimethylaminopyridine (DMAP) and rosiglitazone (RLZ) were purchased from Aladdin (China). Lecithin was supplied by Tokyo Chemical Industry Co., Ltd (Japan). 1,1'-Carbonyldiimidazole (CDI) was provided by Acros Organics (USA). 1,2-Distearoyl-sn-glycero-3-phosphoethanolamine-N-[methoxy(polyethylene glycol)-2000] (DSPE-PEG2000) was obtained from Corden Pharma (Switzerland). Dextran sulfate sodium (DSS, Mw = 35 kDa) was supplied by MP Biomedical (USA). Mouse IL-6 (EMC004.96), IL-1β (EMC001b.96), TNF-α (EMC102a.96) and IL-10 (EMC005.96) ELISA kits were supplied by NeoBioscience (Shenzhen, China). Fetal bovine serum (FBS), penicillin and streptomycin were obtained from HyClone (USA). Dulbecco's Modified Eagle Medium (DMEM) and RPMI1640 medium were supplied by Gibco (USA). MPO assay kit was purchased from Nanjing Jiancheng Bioengineering Institute (Nanjing, China). Nitric oxide assay kit (S0021), superoxide assay kit (S0060), hydrogen peroxide assay kit (S0038) and fluorescence dye (Cyanine5 (Cy5) and Cyanine7.5 (Cy7.5)) were purchased from Beyotime Biotechnology (China). Calcein-AM/PI double stain kit was provided by Solarbio Life Sciences (China). PE-F4/80 (565410), Alexa Fluor^®^ 647-CD206 (565250), APC-CD11b (553312), FITC-CD86 (561962) antibodies were provided by Becton Dickinson (USA). Lipopolysaccharide (L4391) and 2',7'-dichlorofluorescin diacetate (DCFH-DA) were purchased from Sigma-Aldrich. Recombinant murine IL-4 (214-14) was obtained from Peprotech Inc. Transwell insert plates (0.4 μm pore) were purchased from Corning (USA).

^1^H NMR (600 MHz) spectrum was recorded with Bruker Ultra Shield 600 PLUS NMR spectrometer. Flow cytometry analysis was conducted with BD LSR Fortessa cytometer. Tecnai G20 TEM (FEI. Co., USA) was used for transmission electron microscopy (TEM) photography. The dynamic light scattering (DLS) measurement was performed with Zetasizer (Malvern. Co., UK). Fluorescent imaging was performed with Leica TCS SP8 confocal laser scanning microscope (Leica, Germany).

RAW264.7 and Caco-2 cell lines were supplied by American Type Culture Collection (ATCC, Shanghai, China).

### Synthesis of ROS-responsive β-CD (Ox-CD)

The synthesis of Ox-CD was performed according to a previously reported method 28. Briefly, PBAP and CDI were dissolved in dry dichloromethane, and the mixture was stirred overnight. The mixture was subsequently washed with deionized water. The organic phase was collected and washed with saturated NaCl solution, and was subsequently dried overnight and concentrated in vacuum to obtain CDI-PBAP. Next, β-CD was dissolved in anhydrous DMSO and DMAP, which was added CDI-PBAP. The mixture was stirred overnight. The final product was obtained by precipitation in water and collected by centrifugation. The structure of synthesized Ox-CD was confirmed by ^1^H NMR spectroscopy, consistent with the previous report 29.

### Fabrication of Ox-CD NPs

The fabrication of Ox-CD NPs was based on the reported literature method 29. Briefly, the synthesized Ox-CD and payload (RLZ or Cy5 or Cy7.5) were dissolved in anhydrous methanol and acetonitrile (1:1). Lecithin and DSPE-PEG2000 were dissolved in ethanol and then deionized water was added. The dispersion was heated to 65 °C and kept stirred for 0.5 h. The Ox-CD solution was added dropwise into the DSPE-PEG dispersion under ultrasonication, followed by magnetically stirring for 2 hours to allow evaporation of the organic solvents. Finally, vacuum evaporation was utilized to remove the remaining organic solvent and water, and Ox-CD NPs were obtained.

### Isolation of membrane from RAW264.7 cells

Macrophage membrane was isolated from RAW264.7 cells based on a literature approach 20. Specifically, RAW264.7 cells were harvested and washed three times with PBS. The cell pellet was suspended in TM buffer containing 0.01 M Tris and 0.001 M MgCl_2_ (pH = 7.4). The suspension was incubated in ice-bath for 20 minutes and was subsequently extruded through a mini-extruder (Avestin, LF-1, Canada) for 20 times for cell lysis. Next, 1 M sucrose TM buffer was added to make a final concentration of 0.25 M sucrose TM buffer. The mixture was centrifuged at 2000 g at 4 °C for 10 min to remove the nucleus pellet. The supernatant was collected and further centrifuged at 3000 g at 4 °C for 30 min. The resultant pellet (cell membrane) was collected and washed with TM-buffer (containing 0.25 M sucrose) and centrifuged again at 3000 g at 4 °C for 30 min. The protein content in the derived membrane was determined by BCA protein assay.

### Preparation and characterization of membrane coated NPs

The membrane coating was realized by extrusion. Briefly, Ox-CD NPs were mixed with the purified macrophage membrane (at a weight ratio of 1:1 NPs: membrane protein) and then extruded through a polycarbonate membrane with pore size of 400 nm to afford membrane coated NPs. The particle size and morphology of membrane coated NPs were analyzed by TEM. The particle size and zeta potential were further determined by DLS. With regards to the stability, the NPs were stored at 4 °C in deionized water or PBS and the size and zeta potential were determined at different time points. The protein profiles of the macrophage membrane coated NPs were analyzed by sodium dodecyl sulphate-polyacrylamide gel electrophoresis (SDS-PAGE).

### *In vitro* drug release assay

10 mg RNs were coated with cell membrane to fabricate RMN and these RMN NPs were added into dialysis bag (MWCO = 3500 Da), which was subsequently immersed in 50 mL PBS containing 0.2 v/v% Tween 80 containing 0, 0.1, 1 mM hydrogen peroxide (H_2_O_2_), respectively. The system was incubated at 37 ºC with a shaking rate of 50 rpm. At each pre-determined time point, 5 mL medium was drawn and the same volume of fresh medium was supplemented into the release medium. The drug concentration in the released medium was analyzed by high performance liquid chromatography (HPLC) and the percentage of drug release is equal to the amount of accumulated rosiglitazone in the medium divided by the total rosiglitazone content in the tested NPs.

### Internalization of nanoparticles

Free Cy5 and Cy5 loaded NPs without membrane coating (Cy5-N) and Cy5 loaded membrane coated NPs (Cy5-MN) (each containing the same content of Cy5) were added into the medium of BMDM and Caco-2 cells with the final concentration of Cy5 as 10 μg/mL and the concentration of NPs as 0.19 mg/mL. The internalization of free Cy5 and Cy5 loaded NPs was determined by flow cytometry in 4 hours and 24 hours, respectively according to the previous studies 30, 31 and the mean fluorescence intensity was statistically analyzed.

### Macrophage polarization assay

Bone marrow derived macrophages (BMDM) were isolated and differentiated according to a previously reported method 32. Briefly, total bone marrow cells were incubated in RPMI 1640 media containing 10% FBS, 1% antibiotics and 20% L929 supernatant. The media was refreshed on Day 4. The cells were harvested on Day 7 and verified with F4/80 and CD11b fluorophore-conjugated antibodies. BMDMs were subsequently stimulated with 100 ng/mL LPS for M1 polarization, or 20 ng/mL IL-4 for M2 polarization, in the absence and presence of free RLZ (10 μM) or RLZ-loaded NPs (various formulations), respectively. M1 macrophage marker CD86 and M2 macrophage marker CD206 were determined by flow cytometry. TNF-α, iNOS, IL-1Rn and Fizz-1 were determined by qPCR.

### ROS, NO and inflammatory cytokine determination

DCFH-DA was utilized to determine the intracellular ROS level in BMDM cells. Specifically, BMDM cells were incubated with 100 ng/mL LPS together with free RLZ or RLZ-loaded NPs (71.5 μg/mL), at RLZ dose of 10 μM for 24 h. The cells were washed and treated with 20 μM DCFH-DA for 20 min. DCFH-DA was subsequently washed out and flow cytometry was used to determine the fluorescence intensity of cells. With regards to the nitric oxide (NO) content in the supernatant, Griess reagent was utilized to determine NO quantity, following the manufacture's instruction. Inflammatory cytokines in the supernatant were determined with ELISA kits.

### Cell viability assay

BMDM cells or Caco-2 cells were incubated in normal media or H_2_O_2_ (10 mM) containing media according to a previous literature 33, and RLZ, RN, RMN or Mem (cell membrane liposomes) was respectively added with the final concentration of RLZ as 10 μM (the corresponding concentration of NPs was 71.5 μg/mL). The viability of cells was determined with CCK8 in 24 hours.

### Cell apoptosis assay

Caco-2 cells were incubated in H_2_O_2_ (10 mM) containing media 33 and co-cultured with RLZ or RMN. Apoptosis kit was used to stain the cells after 24 hours and cell apoptosis rate was analyzed *via* flow cytometry.

### Live/dead cell staining

RAW264.7 cells were incubated with RLZ, RN or RMN (with the same concentration of RLZ as 10 μM) for 24 hours and then stained with calcein-AM (2 μM) and propidium iodide (PI, 5 μM) for 30 min, followed by imaging with confocal laser scanning microscopy. The percentage of live cells was quantified from 6 independent images.

### Cell proliferation assay

5×10^5^ RAW264.7 cells were incubated with RLZ, RN or RMN (with the same dosage of RLZ as 10 μM) for 48 hours and the cell numbers were counted at 24 h and 48 h time points.

### Caco-2 cells co-cultured with RAW264.7 cells

Caco-2 cells were seeded onto 24-well plate. RAW264.7 cells were firstly seeded onto transwell inserts (0.4 μm pores) and then transferred to 24-well plate after adhesion for co-culturing with Caco-2 cells. A group without RAW264.7 cells in the transwell inserts was set as control. Different formulations of RLZ (RLZ, RN, RMN, containing the same dosage of RLZ as 10 μM) were added to the apical side of transwell inserts together with 100 ng/mL LPS. The viability of Caco-2 cells was determined via CCK8 assays in 24 hours. RAW264.7 cells were collected and stained with fluorophore labeled CD86 and CD206 antibodies and these markers were determined by flow cytometry.

### Induction of colitis and treatment protocol

Male C57BL/6 mice (6 weeks) were supplied by the Animal Facility, Faculty of Health Sciences, University of Macau. Animals were housed under standard conditions of temperature, light, and humidity, with ad libitum access to water and food. The animal experiment protocol was approved by the Animal Ethics Committee, University of Macau. Colitis in mice was induced by giving 3% (w/v) DSS water for 7 days and during the 7- day colitis induction period, different formulations of RLZ were administered. Mice were randomly divided into 6 groups (6 mice in each group) including the normal control group (without DSS treatment) treated with saline, and five colitis groups respectively treated with saline, free RLZ, RLZ loaded Ox-CD NPs (RN), blank membrane coated Ox-CD NPs (MN), and RLZ-loaded membrane coated Ox-CD NPs (RMN) with all formulations dissolved or suspended in saline. All treatment groups were given the same amount of RLZ (10 mg/kg) 34 or blank NPs intravenously *via* tail vein on Day 0, 3 and 5 (three times totally) according to a modified literature treatment plan 35 with the first injection set on Day 0 (6 hours after the initial DSS treatment) to exploit the therapeutic potential of RLZ at the early stage of inflammation 36. At the same time, the injection frequency was increased accordingly as the inflammation was aggravated over time. Intravenous administration was adopted for protection of membrane proteins, which are essential for maintaining long systemic circulation, inflammatory tropism and sequestration of inflammatory cytokines 12.

### *Ex vivo* distribution of NPs in mice

Cy7.5 loaded NPs with or without cell membrane coating were used to evaluate their biodistribution in DSS-induced colitis mice *in vivo* and compared with that of free Cy7.5. Nine mice were fed with 3% (w/v) DSS water for 5 days and then randomly divided into 3 groups (3 colitis mice in each group), which were respectively administered *via* tail vein with Cy7.5, Cy7.5-N, Cy7.5-MN with the same dosage of Cy7.5 (1 mg/kg). The major organs including the heart, livers, spleen, lungs, kidneys and colon were isolated at 1, 3, 6, 9, 12 h after intravenous injection of free Cy7.5 or Cy7.5 loaded NPs. The isolated organs were imaged *ex vivo* by living imaging system (IVIS Lumina III *In Vivo* Imaging System). The fluorescence intensity in different organs was analyzed with Living Imaging^®^4.3.1 software.

### Therapeutic efficacy evaluations

During the 7-day treatment of colitis, three parameters (body weight, rectal bleeding and stool consistency) were daily examined and recorded. The disease activity index (DAI) was measured as the sum of these three indexes, including body weight loss index (0-4), stool consistency index (0-3) and rectal bleeding index (0-3). Mice were sacrificed at the end of the experiment. The weight of the spleen and the length of the colon were measured. And the colons were harvested and excised after thorough wash. Myeloperoxidase (MPO) activity assay, determination of inflammatory cytokines, quantification of hydrogen peroxide and superoxide were performed following the manufactures' instructions. A separated part of colon was used for the determination of the mRNA levels of TNF-α, IL-1β, IL-6 and IL-10 by qPCR.

### Biocompatibility study

Normal male C57BL/6 mice were divided into two groups (3 mice in each group), intravenously administered with saline and RMN, respectively. Saline or RMN was given three times (Day 0, Day 3 and Day 5). On the 7^th^ day, Mice were sacrificed and all major organs (including the heart, livers, spleen, lungs, and kidneys) and the colon were collected for histological analysis. Blood was collected for hematology assays as well as liver and kidney function biomarkers assessment.

### Histological analysis of colon and major organs by hematoxylin and eosin staining

Major organs and the colon were fixed in 4% paraformaldehyde for 24 h before embedded in paraffin. After sectioned at 4 μm thickness, all organs were stained with hematoxylin and eosin (H&E). Images were photographed with a Nikon microscope equipped with a digital camera. Histology of the colon was graded with a scoring system according to the reference 37. Briefly, inflammation severity (0-3), depth of injury (0-3), crypt damage (0-4) and percentage of the area involved (0-4) were assessed and the sum was presented as the histological score.

### Immunohistochemistry assay of the colon tissues

The colon sections in paraffin were deparaffinized and immersed in 0.3% H_2_O_2_-PBS buffer for 1 h to eliminate the interference of endogenous peroxidase. After washed three times, the sections were blocked in 10% bovine serum albumin containing 0.25% Triton X-100. Antibodies against inducible nitric oxide synthase (iNOS), CD86, arginase-1 and CD206 were implemented to mark relevant proteins, respectively. After secondary antibody incubation and substrate reaction, the final immunohistochemistry analysis was carried out with Nikon microscope.

### PAS staining of colon tissues

Colon sections embedded in paraffin were stained with PAS (periodic acid-Schiff) and then photographed with a digital camera.

### Statistical analysis

Data are presented as mean ± S.D. One-way ANOVA was performed for statistical analysis and a value *p* < 0.05 was considered statistically significant.

## Results and Discussion

### Preparation and Characterization of RMN NPs

**Figure 1:**
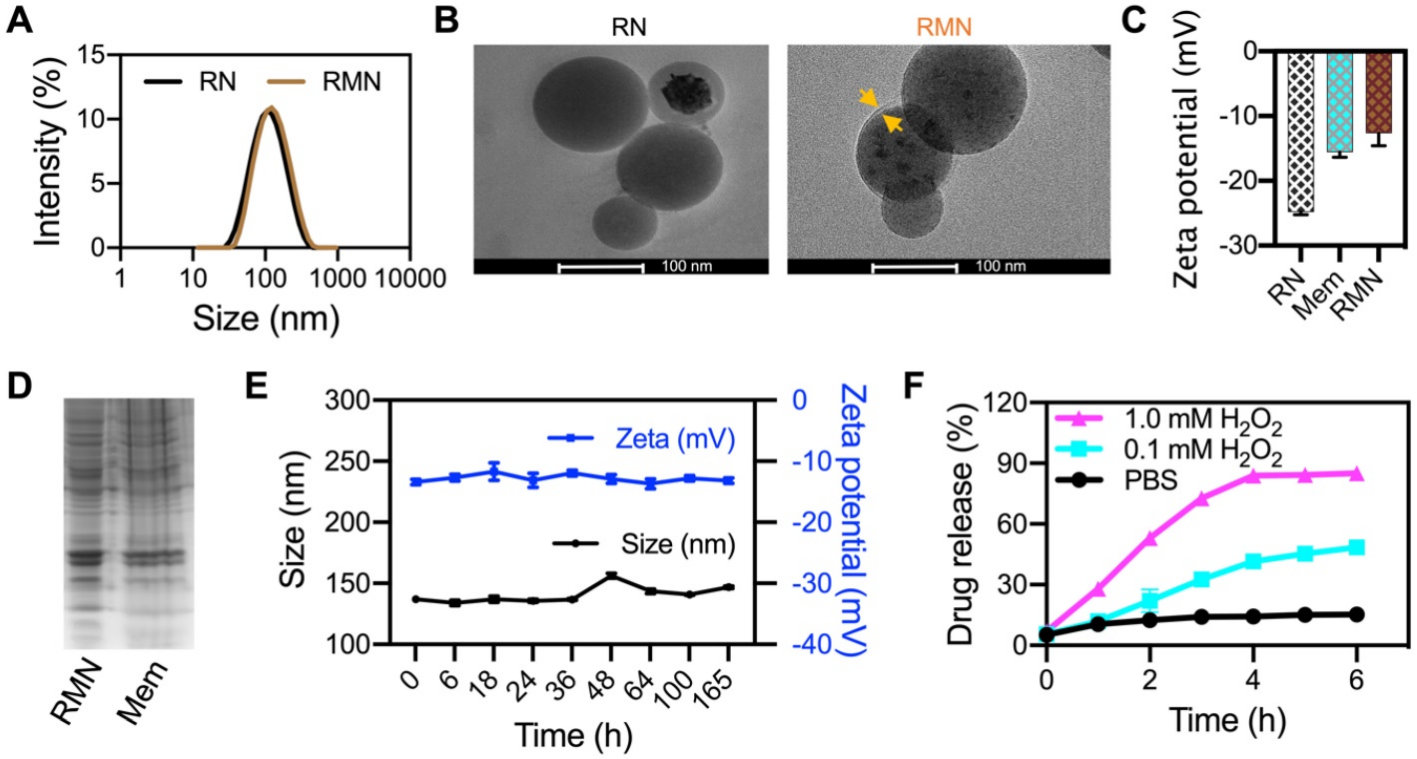
** Characterizations of NPs.** (**A**) Size distribution of RN and RMN NPs measured *via* DLS. (**B**) TEM images of RN and RMN. Coated membrane layer was indicated by yellow arrows. (**C**) Zeta potentials of NPs. (**D**) Protein profiles of RMN NPs and Mem by SDS-PAGE. (**E**) The size and zeta potentials of RMN stored in H_2_O at 4 °C for up to 7 days. (**F**) Drug release kinetics from RMN, incubated with 0, 0.1, and 1.0 mM H_2_O_2_ in PBS. Data are shown as mean ± S.D. (*n* = 3).

The structure of synthesized Ox-CD was verified by NMR spectroscopy and FTIR (Figure S1). RLZ loaded NPs (RNs) were fabricated with the assistance of DSPE-PEG2000 and lecithin, according to a literature method 29. The drug encapsulation efficiency (DEE) and drug loading content (DLC) of RNs were 56.8% and 5.3%, respectively, determined by HPLC. RMN NPs were prepared by coating RLZ loaded Ox-CD NPs (RNs) with RAW264.7 cell membrane. The mean diameter of RMN NPs was 135.2 nm, approximately 22 nm larger than that without membrane coating, as was determined by DLS (Figure 1A). TEM analysis (Figure 1B) revealed spherical morphology of both RN and RMN with mean diameters of ca. 95 nm and 100 nm, respectively, moderately smaller than the sizes observed *via* DLS, likely attributed to the hydration effect of NPs in water during DLS measurements. In addition, the membrane shell coating was evidenced in the TEM images of RMN, with a thickness of ca. 6 nm, similar to the thickness of cell membrane of ca. 7.5-10 nm 38. The zeta potential of RMN was modestly less negative than that of RN, but rather similar to that of membrane liposomes (Mem) (Figure 1C), suggesting the similar surface properties between RMN and Mem. Moreover, most of the proteins in cell membrane (Mem) were well conserved in the final RMN, observed from the profiles of SDS-electrophoresis (Figure 1D), suggesting the successful coating of macrophage membrane on Ox-CD NPs. In addition, these NPs were stable under storage conditions (4 °C), as their size and zeta potential (Figure 1E and Figure S2A) in deionized water remained stable for up to 1 week. RMN were also relatively stable in PBS buffer (Figure S2B) 39. Subsequently, the ROS-responsiveness of RMN was investigated under different concentrations of H_2_O_2_. As shown in Figure 1F, the payload was released in a ROS-level dependent manner, and RMNs exhibited efficient ROS responsiveness even with membrane coating.

### RMN NPs polarized macrophages to M2 and protected epithelial cells from oxidative stress

**Figure 2:**
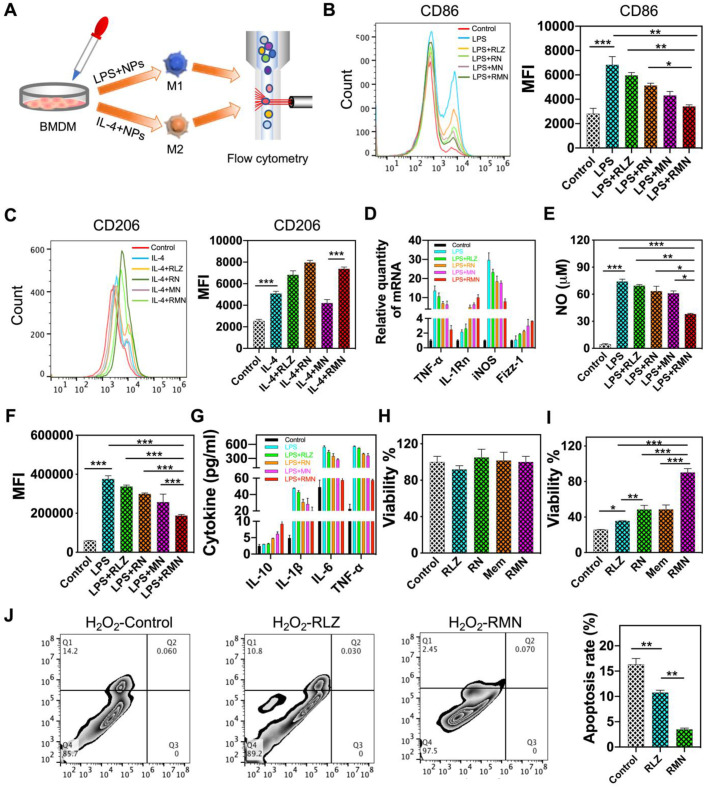
** RMN polarized macrophages to M2 and protected Caco-2 cells from oxidative stress.** (**A**) Scheme showing the experimental approach: BMDM cells were polarized to M1 or M2 and were co-incubated with free RLZ and various NP formulations during the polarization process. The expression of surface markers was determined by flow cytometry. (**B**) CD86 and (**C**) CD206 determined *via* immunofluorescent staining and flow cytometry analysis. (**D**) Relative mRNA levels of TNF-α, IL-1Rn, iNOS and Fizz-1 determined by qPCR. (**E**) NO production in culture supernatant. (**F**) ROS determined by DCFH-DA staining and flow cytometry (with the mean fluorescence intensity statistically analyzed). (**G**) Protein content of cytokines in culture supernatant determined by ELISA. (**H**) Cell viability of Caco-2 cells with different treatments, in the absence and presence of H_2_O_2_. (**I**) Apoptosis of Caco-2 cells in media containing 10 mM H_2_O_2_ and PBS, RLZ and RMN, respectively. Data are shown as mean ± S.D. (*n* = 3). *p < 0.05, **p < 0.01, ***p < 0.001.

As a part of immunological response, a large number of macrophages are recruited and accumulate in the ulcerative colon, resulting in sustained inflammation 40, 41. Under different stimuli, macrophages undergo disparate fates and polarize to one of mainly two phenotypes, M1 and M2. M1 macrophages produce pro-inflammatory cytokines and ROS, whereas M2 macrophages secret anti-inflammatory cytokines, accelerate inflammation resolution and promote tissue repair 42, 43, thus M2 macrophage was previously leveraged for UC therapy 22. As RLZ was found to drive polarization of macrophages to M2 24, 25, it was selected as the therapeutic payload in this study. The scheme in Figure 2A shows the experimental approach assessing the macrophage-polarizing effects of free RLZ and RLZ-loaded NPs *in vitro*. As shown in Figure 2B, bone marrow-derived macrophages (BMDM) treated with LPS increased the expression of CD86, one of the typical markers of M1 phenotype. Co-treatment of macrophages with LPS and free RLZ or RLZ-loaded NPs (RN) decreased the expression of CD86, and RN showed stronger inhibitory effects than free RLZ in CD86 expression, partly due to the better cellular internalization of NPs (Figure S3). Interestingly, MN (blank Ox-CD NPs coated with macrophage membrane, without loading of RLZ) also decreased CD86 to a moderate extent, likely by absorbing inflammatory cytokines, which was previously reported 20. Among all different formulations, RMN exhibited the best efficacy in decreasing CD86, attributed to both the macrophage-polarizing effects of RLZ and the pro-inflammatory cytokine absorbing effects of cell membrane. On the other hand, RLZ enhanced polarization of macrophages to M2 (Figure 2C), as the increased level of CD206, a typical marker of M2, was observed. As expected, RMN and RN exhibited improved M2-polarizing effects than free RLZ. In order to further verify the polarization of macrophages, other M1 markers including TNF-α and iNOS, and M2 markers including IL-1Rn and Fizz-1, were all determined by qPCR. Consistent with the results of CD86 and CD206, RMN remarkably decreased TNF-α and iNOS and increased IL-1Rn and Fizz-1 (Figure 2D). RMN also exhibited the most significant effects in lowering other inflammatory mediators: NO and ROS levels as well as pro-inflammatory cytokines (Figure 2E-G, Figure S4). Concurrently, the anti-inflammatory cytokine IL-10 was effectively increased after these treatments, with RMN standing out as the most effective one. Moreover, we found that RMN increased the viability (Figure 2H) of Caco-2 cells under oxidative stress by reducing H_2_O_2_-induced apoptosis (Figure 2I). Under normal conditions (without H_2_O_2_), RLZ, RN, RMN and Mem did not significantly affect the viability of Caco-2 cells, suggesting the safety of the drug and the carriers in different formulations. However, with H_2_O_2_ mimicking oxidative stress, RLZ, RN, RMN and Mem all improved cell viability, implying the protective role of both membrane and RLZ against ROS induced cell damage, likely attributed to the anti-apoptotic effects of RLZ 44, 45. The coated membrane could sequester H_2_O_2_ induced pro-inflammatory cytokines 46, 47, which would otherwise cause additional injury to colonic epithelial cells. In addition, these three formulations of RLZ (free RLZ, RN and RMN) also exhibited excellent safety profiles in macrophages with negligible influence observed on cell viability (Figure S5 and S6A) and proliferation (Figure S6B). To further investigate the role of macrophage polarization in the regulation of colonic inflammation, RAW264.7 cells and Caco-2 cells were co-cultured in the presence of LPS and different formulations of RLZ. As shown (Figure S7A), the viability of Caco-2 cells was decreased significantly when co-cultured with LPS polarized macrophages that could secrete pro-inflammatory cytokines and cause damage to Caco-2 cells 48. RLZ, RN and RMN all increased the viability of Caco-2 cells by inhibiting the M1 polarization of macrophages (Figure S7B). The increased internalization of RN, when compared to free RLZ, contributed to the better performance of RN over free RLZ, and the sequestration of pro-inflammatory cytokines by the coated membrane of RMN further enhanced the protective effects. Taken together, these results strongly supported that RMN effectively alleviated inflammation *in vitro* and protected colon epithelial cells from oxidative stress.

### Biodistribution and targeted accumulation of RMN in the inflammatory colon

Macrophage membrane coated NPs may accumulate in inflammatory tissues specifically due to their inherent inflammation-homing effects 18. Particularly, the integrin adhesion receptors located in the membrane may assist in the adhesion of NPs to inflamed endothelium 12. Therefore, the biodistribution of NPs was investigated *in vivo*, upon the biosafety of RMN was systemically demonstrated in mice. RMNs were intravenously injected into healthy mice *via* tail vein for safety evaluation. As expected, both the hematological analysis (Figure S8A) and histopathological analysis of major organs (Figure S8B) revealed a decent safety profile of RMN *in vivo*. In addition, no obvious systemic inflammation was observed from the content of inflammatory cytokines in the blood serum (Figure S8C), suggesting that RMN did not induce significant immunogenicity *in vivo*. All these findings demonstrated an excellent safety profile of RMN *in vivo*. For the biodistribution assay, free Cy7.5, Cy7.5 loaded NPs (Cy7.5-N) and Cy7.5 loaded membrane coated NPs (Cy7.5-MN) were respectively injected into colitis mice intravenously and major organs and the colon were collected at different time points (1, 3, 6, 9 and 12 h) for *ex vivo* imaging. As shown in Figure S9A and S9B, Cy7.5 in all formulations mainly accumulated in the liver due to the liver's filtration effects 49. Meanwhile, it was obvious that free Cy7.5 reached peak in the colon at 3 h post administration, but was quickly cleared out afterwards. Both Cy7.5-N and Cy7.5-MN exhibited much better accumulation in the colon than free Cy7.5, and both reached peaks at 6 h after administration. At this time point, Cy7.5-N improved colon distribution by 1.6 folds compared to free Cy7.5 and membrane coating (Cy7.5-MN) further increased the concentration by 1.9 times attributed to the homing effects and the reduced reticuloendothelial system (RES) clearance of macrophage-biomimetic NPs 50. The enhanced targeting efficiency by membrane coating was also validated by total accumulation in the colon represented as the area under curve (AUC) shown in Figure S9C. Taken together, these results demonstrated that macrophage membrane coated NPs improved inflammatory targeting efficiency in the colon *via* intravenous injection and may therefore enhance the therapeutic effects of its payload.

### RMN alleviated DSS-induced colitis in mice

**Figure 3:**
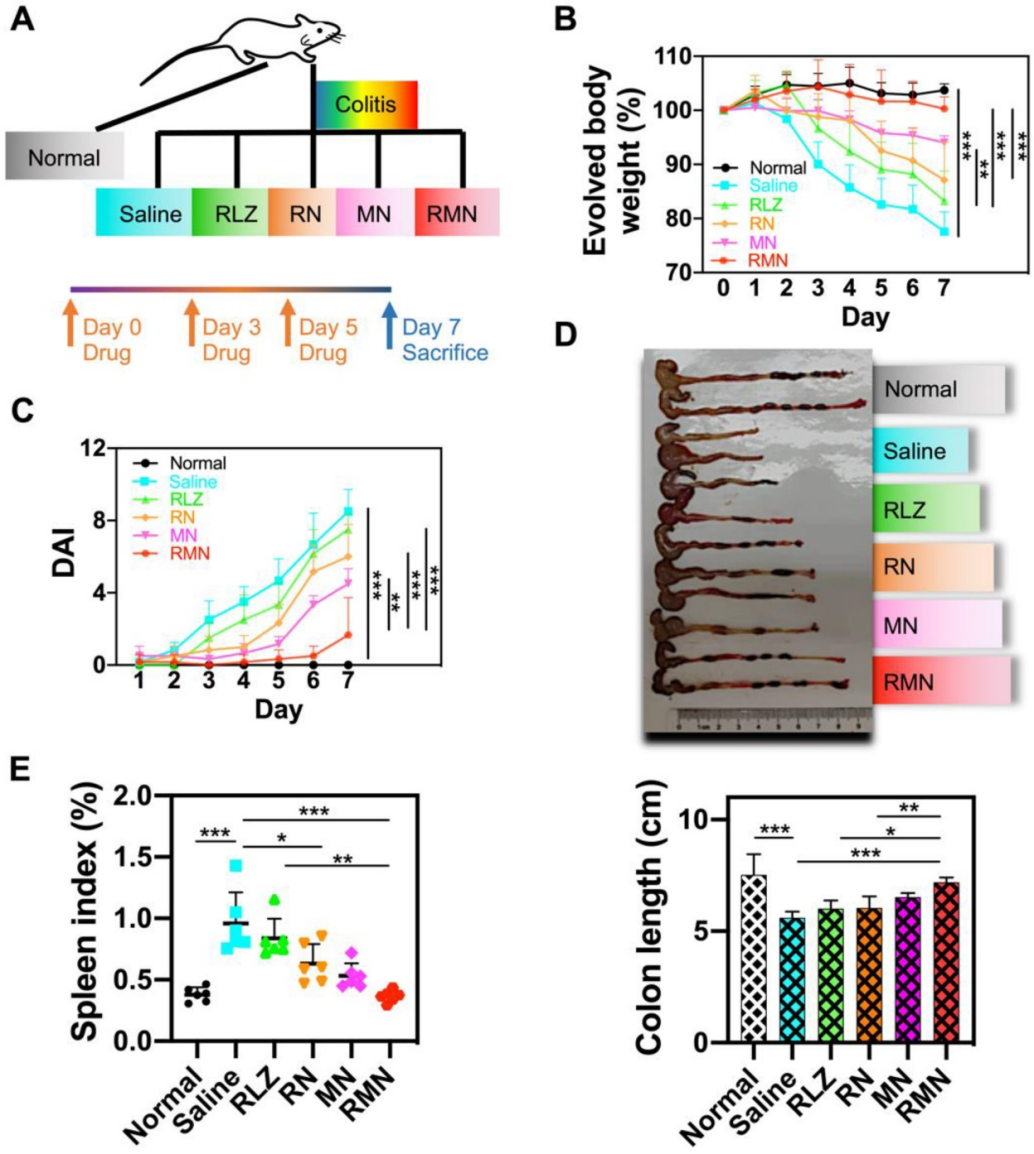
** Therapeutic efficacy in DSS-induced colitis mice model.** (**A**) Experimental protocol of therapeutic treatment of UC in mice. (**B**) Body weight changes of mice (Table S1 for original data). Data were normalized as a percentage of the body weight monitored each day against that on Day 0. (**C**) Disease activity index (DAI), which is the sum of weight loss index, rectal bleeding index and stool consistency index (Table S1 for original data). (**D**) Representative photo of the colons from different treatment groups with statistical analysis. (**E**) Spleen index of mice, represented as a percentage of the spleen weight against each body weight. Data are shown as mean ± S.D. (*n* = 6). *p < 0.05, **p < 0.01, ***p < 0.001.

Dextran sulfate sodium (DSS)-induced colitis mouse model was used for treatment efficacy evaluations of RMN *in vivo*. The experimental protocol was illustrated in Figure 3A. As shown in Figure 3B, the colitis mice treated with saline experienced continuously decreased body weight during the development of colitis, with more than 20% body weight loss by the end of a week. All of other therapeutic groups of mice, respectively treated with free RLZ, RN, MN and RMN, exhibited slower decline of body weight to different extents (by following the order of RMN (slowest) > MN > RN > RLZ) during the treatment. Remarkably, the UC mice treated with RMN had nearly negligible body weight loss. Interestingly, MN, without the therapeutic payload RLZ, also showed impressive therapeutic effects in UC mice against body weight loss, likely attributed to the sequestration of increased pro-inflammatory cytokines. The disease activity index (DAI) (Figure 3C) and colon length (Figure 3D) were also evaluated and both were consistent with the result of body weight evolvement, with RMN showing the strongest therapeutic effects. Moreover, the alleviation of systematic inflammation was also indicated by the spleen index. The saline-treated UC mice had the significantly increased spleen index, suggesting a serious systemic inflammation. All therapeutic groups exhibited alleviated spleen indexes to different levels, with RMN being the most effective in maintaining the spleen index similar to that of the normal control group (Figure 3E), suggesting the extraordinary therapeutic efficacy of RMN against UC in mice.

**Figure 4:**
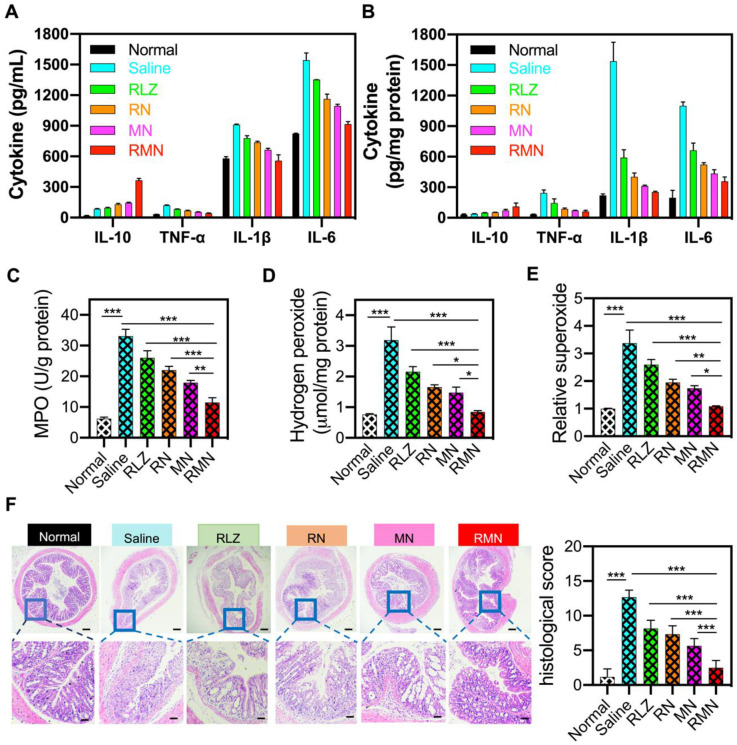
** Inflammatory mediators in the serum and colon and H&E staining of sections from the colons.** Protein levels of inflammatory cytokines in the serum (**A**) and in the colon (**B**). (**C**) MPO activity of colon tissues in different treatment groups. (**D**) Hydrogen peroxide and relative levels of superoxide in the colon tissues. (**F**) Representative H&E-stained sections from the colons in different treatment groups and the histological scores. The scale bars were 50 and 10 microns for the upper and lower panel respectively. Data are shown as mean ± S.D. (*n* = 6). *p < 0.05, **p < 0.01, ***p < 0.001.

During the development of colitis, pro-inflammatory cytokines are generally over-expressed and participate in the pathogenesis of the inflammatory tissues 51. Consistent with the change of mRNAs (Figure S10), the levels of pro-inflammatory cytokines (including IL-1β, TNF-α and IL-6) were decreased and the level of anti-inflammatory cytokine (such as IL-10) was increased in the blood serum and the colonic sites of all treated mice (Figure 4A-B), in comparison with those of the colitis group (treated with saline), suggesting the inflammation in both the circulatory system and colon was suppressed by these treatments, with RMN exhibiting the most pronounced effects. Meanwhile, myeloperoxidase (MPO), a biomarker for neutrophil infiltration 52, 53, increased significantly in the inflammatory colon tissues of the colitis control mice (Figure 4C), indicating that a large number of neutrophils infiltrated into the colon. RMN dramatically decreased the MPO level, with much more pronounced effects than RLZ, RN and MN. Similarly, the hydrogen peroxide (H_2_O_2_) and superoxide levels, as direct indicators of ROS, in the RMN treated group were also the lowest compared to other colitis groups (Figure 4D-E). Moreover, the therapeutic effects against UC in mice were also investigated *via* examination of the histopathological changes of the colons. As shown in Figure 4F, H&E-stained colon sections revealed the serious infiltration of neutrophils, extensive mucosa damage and disappearance of crypts in the colons of UC mice. Inflammation was found in the mucosa, lamina propria and submucosa leaving nearly no intact crypts. RLZ, RN, MN and RMN all improved the histopathological conditions, as was exhibited by alleviated inflammatory severities, shortened injury depth, improved status of crypts and reduced damage area. Among the colitis treatment groups, RMN-treated group of mice showed the best histological conditions in the colon with very similar histological state to that of normal group with only a few crypts injury, as was seen from the histological scores. Taken together, RMN exhibited the best therapeutic efficacy against UC in mice.

### RMN regulated macrophage polarization and protected mucosa *in vivo*

**Figure 5:**
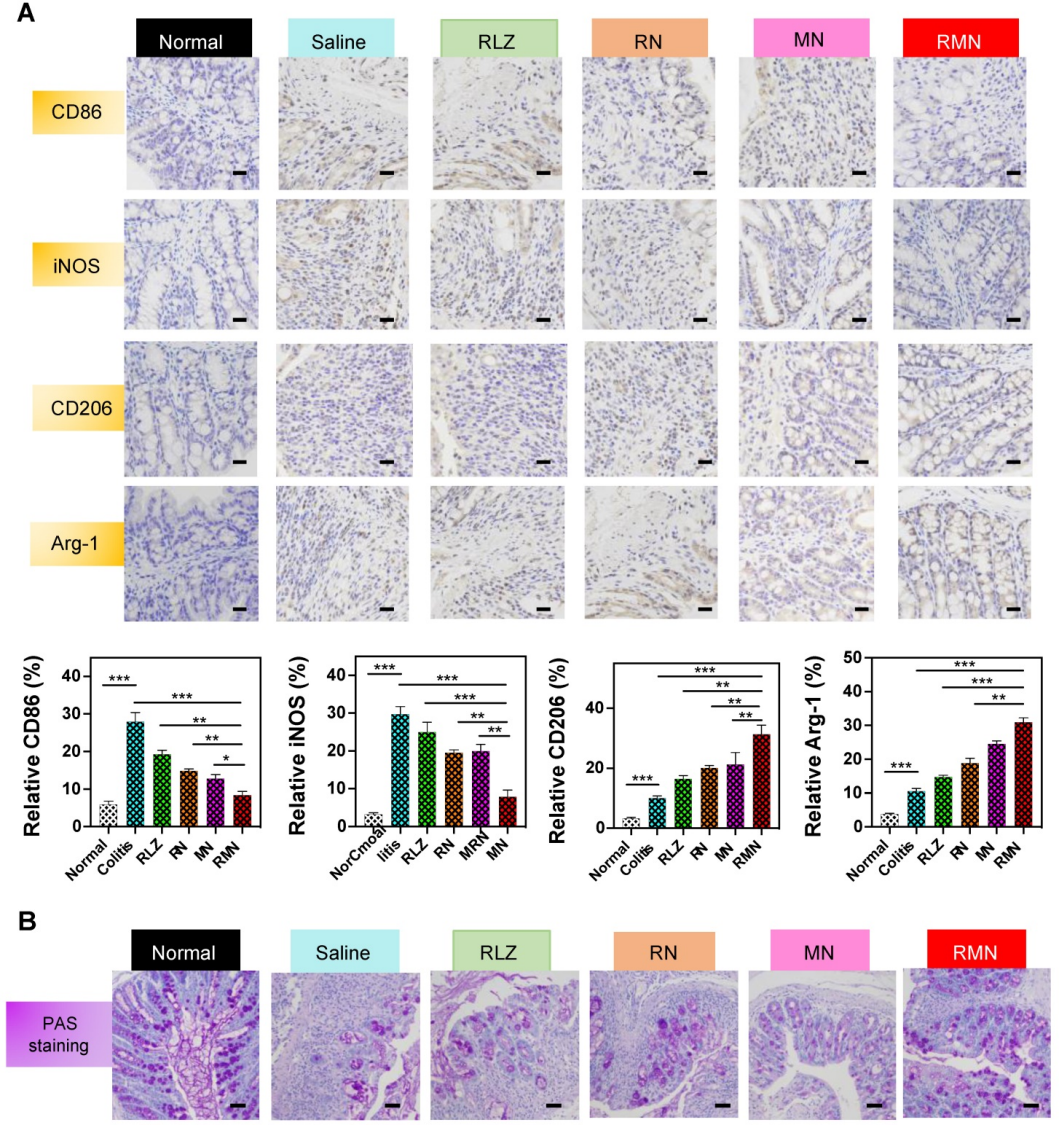
** Immunohistochemical staining of sections from the colons in different treatment groups.** (**A**) Representative IHC staining of CD86, iNOS, CD206 and Arg-1 in the colon tissues and statistical analysis of the results. The scale bars are 10 microns. (**B**) Representative PAS staining of the colons with the scale bars of 5 microns. Data are shown as mean ± S.D. (*n* = 6). *p < 0.05, **p < 0.01, ***p < 0.001.

In order to investigate the role of RMN in regulating macrophage polarization *in vivo,* CD86, iNOS, CD206 and Arginase-1 (Arg-1) in the colon of treated mice were determined by immunohistochemical method (IHC). As shown in Figure 5A, the levels of both CD86 and iNOS were decreased in RLZ and NPs-treated groups compared to those in the saline-treated group, with RMN exhibiting the most remarkable effect. In contrast, the levels of CD206 and Arg-1 in the colon tissues were increased after treatment with different formulations of RLZ, and the increasing extent of CD206 and Arg-1 followed the sequence of RMN>MN>RN>RLZ, consistent with the therapeutic outcomes shown in Figure 3. The decrease of CD86 and iNOS (M1 markers) and simultaneous increase of CD206 and Arg-1 (M2 markers) in the colon tissues indicated the transformation of macrophages from M1 to M2 *in vivo*. Furthermore, the protective effect of M2 macrophages was also verified by the increased number of goblet cells in the colon tissues (Figure 5B). The quantity of goblet cells in the colon of RMN-treated mice was best preserved in comparison to other treatment groups, suggesting that RMN markedly protected mucosa from inflammation-induced damage. Taken together, these results demonstrated that RMN may effectively polarize macrophages to M2 in the inflammatory tissues and protect intestinal mucosa *in vivo*.

## Conclusions

Macrophage-biomimetic nanomedicine was designed and developed based on the pathological characteristics of ulcerative colitis. Attributed to the inflammatory homing effects, macrophage membrane leads the nanomedicine to the inflammatory colonic tissues, where it sequesters inflammatory mediators and suppresses inflammation. Subsequently, in response to the local high ROS level, the nanomedicine releases the therapeutic payload rosiglitazone, which polarizes locally accumulated macrophages to M2, thereby further regulating the inflammatory environment. With the synergistic effects and the excellent safety profile, rosiglitazone loaded macrophage-biomimetic nanomedicine significantly alleviates the colonic inflammation in mice with ulcerative colitis. This novel nanomedicine platform, combining biomimetically-driven targeted delivery and stimuli-responsive release of a therapeutic payload for effective, synergistic therapy of colitis, may shed light on the management of colitis and other inflammatory diseases *via* regulating inflammation.

## Supplementary Material

Supplementary figures and tables.

## Abbreviations

UC: ulcerative colitis
DSS: dextran sulfate sodium
NP: nanoparticle
RBC: red blood cell
WBC: white blood cell
PLT: platelet
HGB: hemoglobin
ALT: alanine aminotransferase
AST: aspartate aminotransferase
CREA: creatinine
LPS: lipopolysaccharide
RLZ: rosiglitazone
Ox-CD: ROS-sensitive β-cyclodextrin
β-CD: β-cyclodextrin
AUC: area under curve
DAI: disease activity index
MPO: myeloperoxidase
Arg-1: arginase-1
iNOS: inducible nitric oxide synthase
IHC: immunohistochemistry
RES: reticuloendothelial system
BMDM: bone marrow-derived macrophage
PAS: periodic acid-Schiff
H&E: hematoxylin and eosin
RN: rosiglitazone loaded Ox-CD nanoparticle
MN: blank membrane coated Ox-CD nanoparticle
RMN: rosiglitazone-loaded and membrane coated Ox-CD nanoparticle
NO: nitric oxide
DLS: dynamic light scattering
TEM: transmission electron microscopy
PLGA: poly(lactic-co-glycolic acid)
SDS-PAGE: sodium dodecyl sulphate-polyacrylamide gel electrophoresis
ROS: reactive oxygen species
DEE: encapsulation efficiency
DLC: drug loading content
HPLC: high performance liquid chromatography
PDI: polydispersity index
Cy5: Cyanine5
Cy7.5: Cyanine5
FTIR: Fourier-transform infrared spectroscopy
PI: propidium iodide
