# Supplementary material for: Amelioration of ulcerative colitis via inflammatory regulation by macrophage-biomimetic nanomedicine
Source: Theranostics. 2020 Aug 8;10(22):10106–19. doi: 10.7150/thno.48448 (PMC7481413; doi:10.7150/thno.48448)
Supplement: Supplementary file 1 — Supplementary figures and tables. [file thnov10p10106s1.pdf]

Supplementary Material

Amelioration of ulcerative colitis via inflammatory regulation by macrophage-biomimetic nanomedicine

Tianlei Sun[1], Cheryl H. T. Kwong[1], Cheng Gao[1], Jianwen Wei[1], Ludan Yue[1], Jianxiang Zhang,[3] Richard Dequan Ye[2,] * and Ruibing Wang[1,] *

[1] State Key Laboratory of Quality Research in Chinese Medicine, Institute of Chinese Medical Sciences, University of Macau, Taipa, Macau, 999078, China.

[2] School of Life and Health Sciences, The Chinese University of Hong Kong, Shenzhen, Guangdong, 518172, China.

[3] Department of Pharmaceutics, College of Pharmacy, Third Military Medical University (Amy Medical University), Chongqing, 400038, China.

*Corresponding address: rwang@um.edu.mo, and richardye@cuhk.edu.cn.

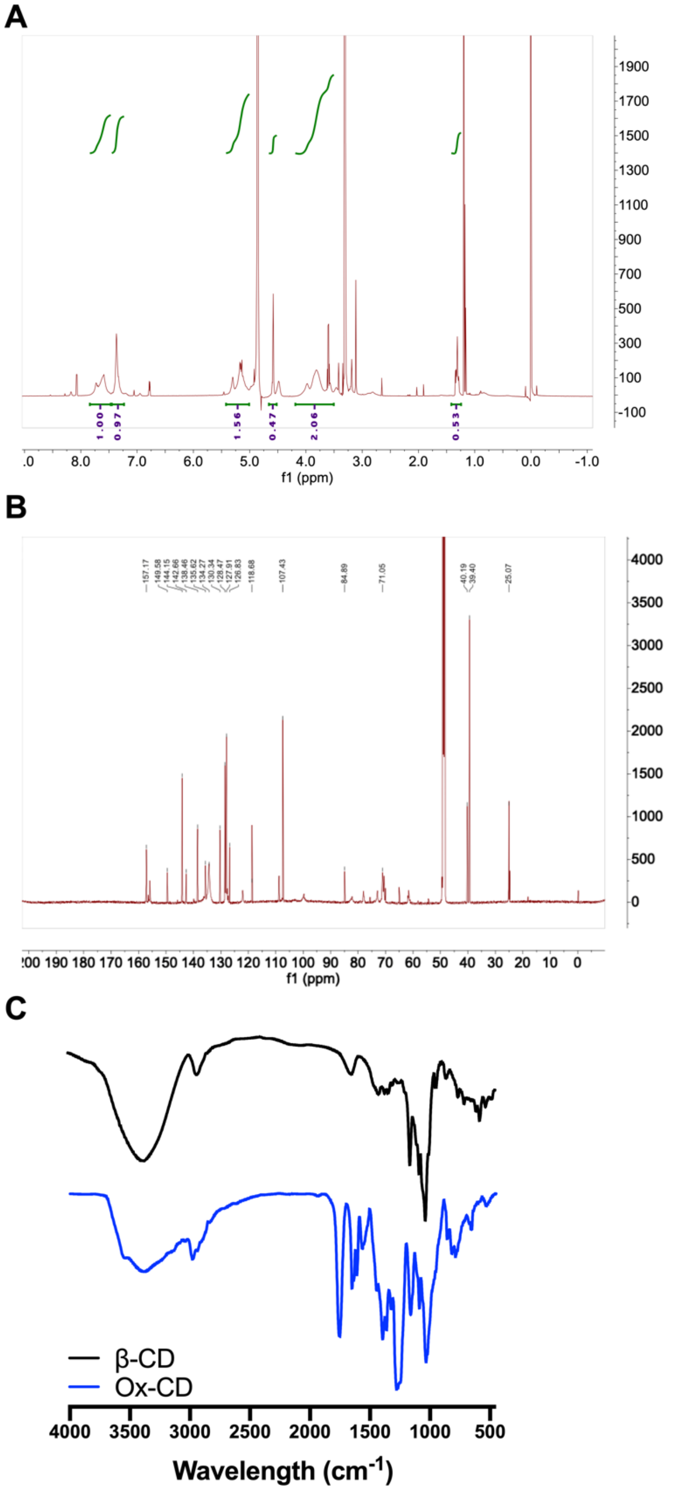

Figure S1. Characterizations of synthesized Ox-CD. (A) ^{1}H NMR spectrum of Ox-CD in CD_3OD (600 MHz). (B) ^{13}C NMR spectrum of Ox-CD in CD_3OD (600 MHz). (C) FTIR spectrum of β-CD and Ox-CD.

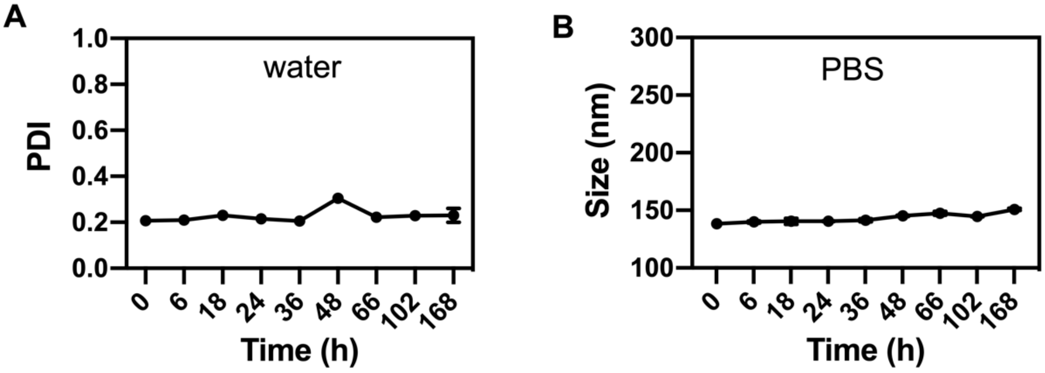

Figure S2. Storage stability of RMN. Size stability (by PDI) of RMN in deionized water (A) and stability of RMN in PBS determined by nanoparticle size (B). Data are shown as mean ± S.D. (n = 3).

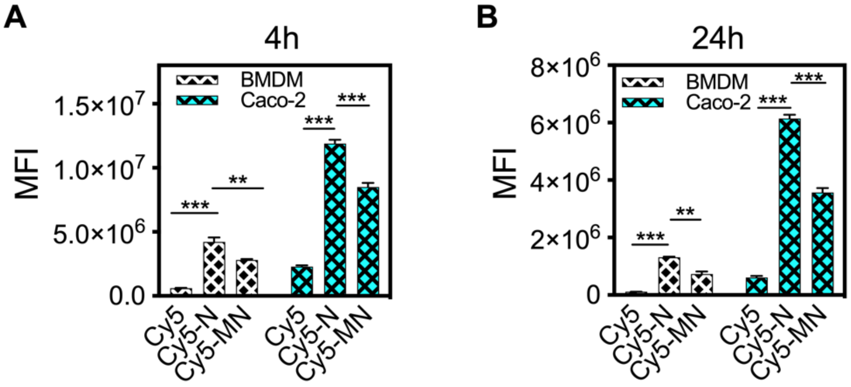

Figure S3. Internalization of nanoparticles in BMDM and Caco-2 cells. BMDM or Caco-2 cells were incubated with free Cy5, Cy5 loaded NP (Cy5-N) or Cy5 loaded and membrane coated NP (Cy5-MN) and the internalization was determined at 4 h (A) and 24h (B) by flow cytometry. Data are shown as mean ± S.D. (n = 3). **p < 0.01, ***p < 0.001.

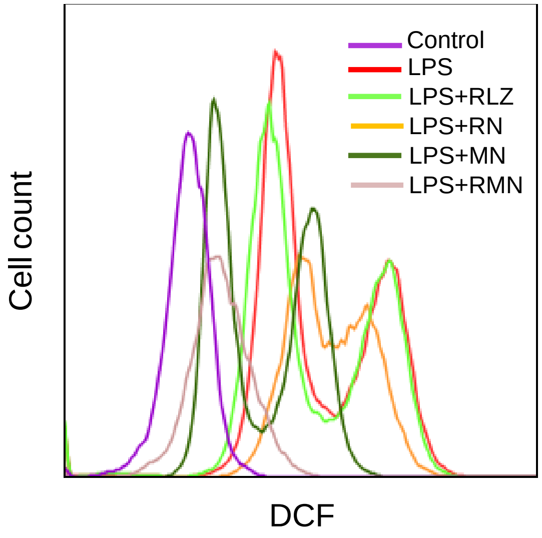

Figure S4. Flow cytometry result in the determination of intracellular ROS. BMDM cells were treated with LPS (100 ng/ml) and different formulations of RLZ (10 μM) for 24 hours and then cells were stained with DCFH-DA and the ROS level was determined by flow cytometry. The graph is representative of 3 independent experiments.

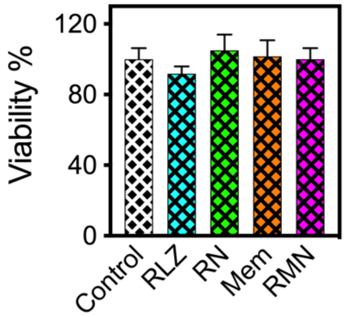

Figure S5. Cell viability of BMDM cells with different treatments. BMDM cells were treated with RLZ, RN or RMN (all containing the same dosage of RLZ as 10 μM) for 24 hours and the viability of cells was determined via CCK8 assays. Data are shown as mean ± S.D. (n = 3).

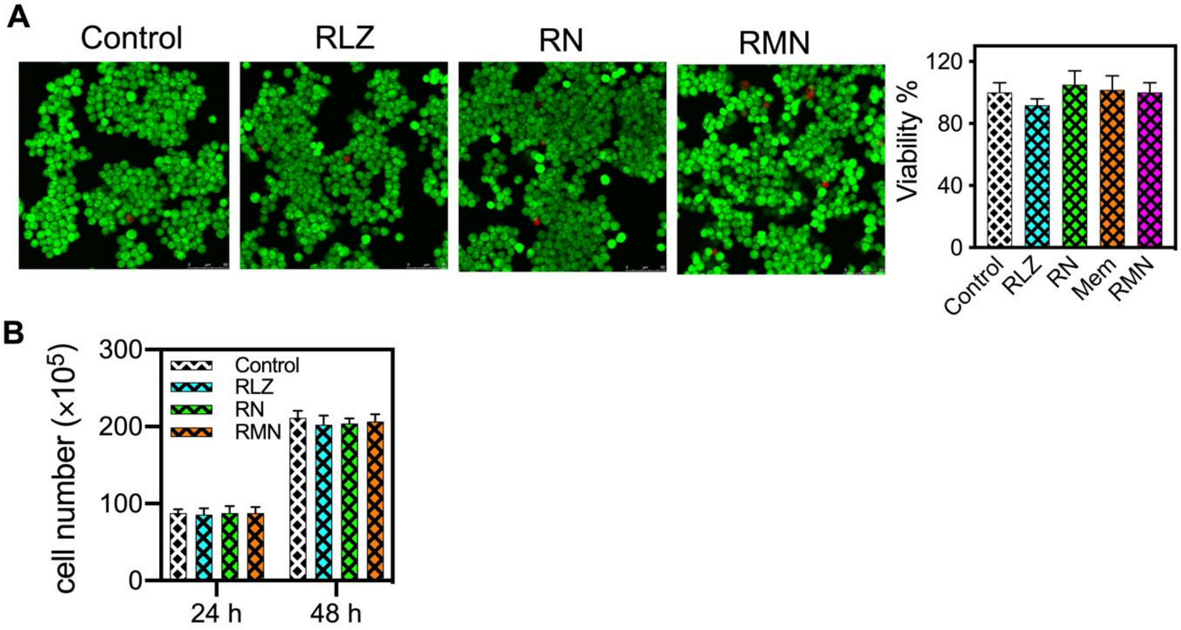

Figure S6. Effects of different formulations of RLZ on the viability and proliferation of RAW264.7 cells. (A) RAW264.7 cells were treated with RLZ, RN or RMN (with the concentration of RLZ as 10 μM) for 24 hours and then cells were stained with calcein AM (green fluorescence) and PI (red fluorescence). The fluorescent images of cells were taken with confocal laser scanning microscope and the proportion of live cells was quantified with 6 independent images (*n* = 6). (B) 5×10^5 RAW264.7 cells were incubated with RLZ, RN or RMN (with the same dosage of RLZ as 10 μM) for 48 hours and the cell numbers were counted at 24 h and 48 h time points (*n* = 3). Data are shown as mean ± S.D.

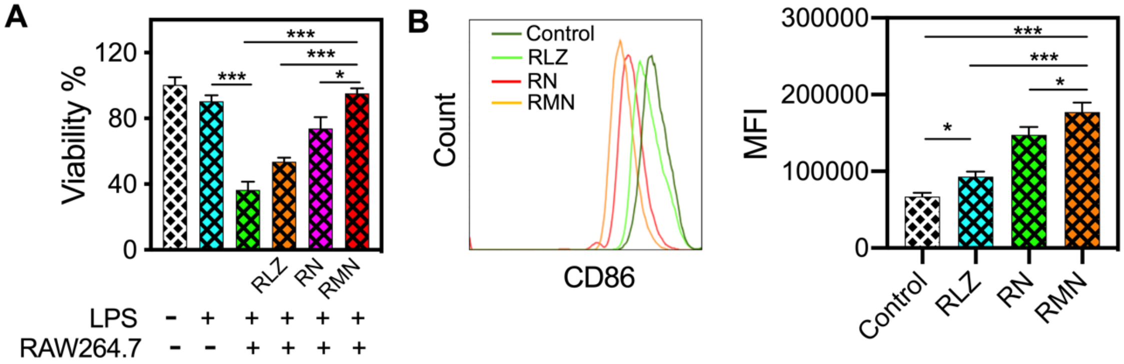

Figure S7. Viability of Caoc-2 cells and polarization of macrophages in the co-culture system under inflammatory condition induced by LPS. Caco-2 cells were co-cultured with RAW264.7 cells and different formulations of RLZ were added into the media together with 100 ng/mL LPS. (A) The viability of Caco-2 cells determined in 24 hours. Data were statistically analyzed with two-way ANOVA. Data are shown as mean ± S.D. (n = 3). *****p < 0.05, *******p < 0.001. (B) Expression of CD86 markers on RAW264.7 cells. The flow cytometry graph was representative of 3 independent experiments and the mean fluorescence intensity (MFI) was statistically analyzed. Data are shown as mean ± S.D. (n = 3). *****p < 0.05, ******p < 0.01, *******p < 0.001.

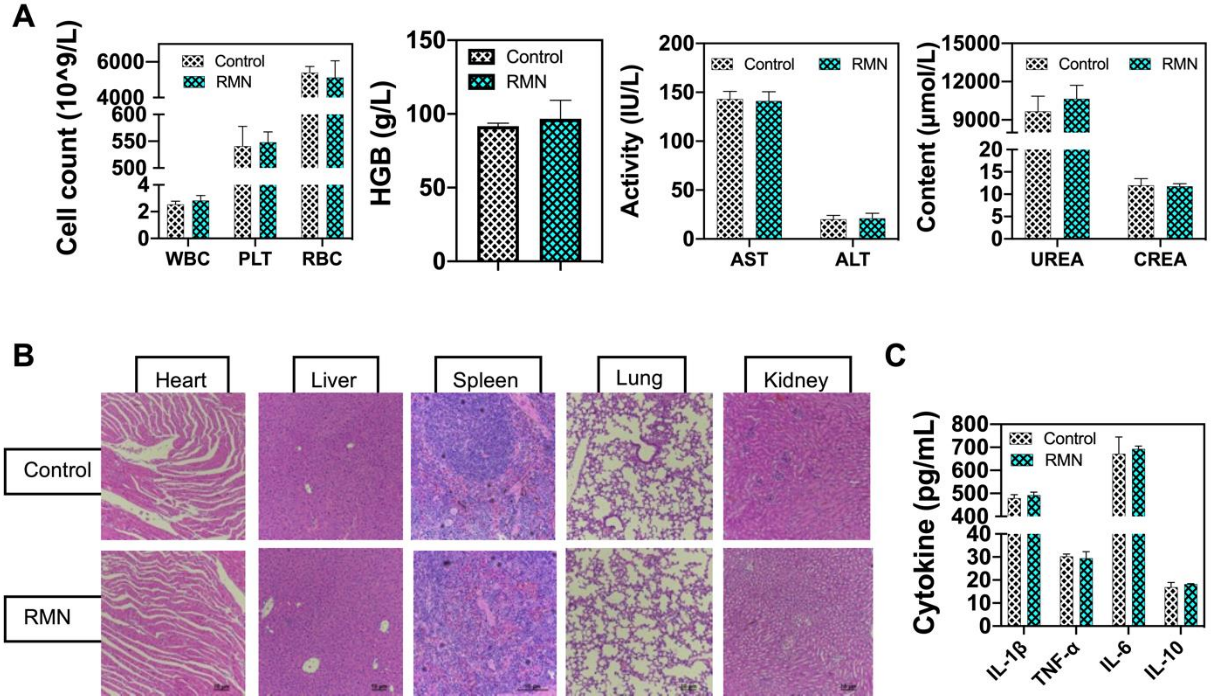

Figure S8. Safety of RMN *in vivo*. (A) Hematological parameters including white blood cells (WBC), red blood cells (RBC), platelets (PLT) counts and hemoglobin (HGB) content as well as biomarkers relevant to the liver and kidney functions, including alanine aminotransferase (ALT), aspartate aminotransferase (AST), blood urea (UREA) and creatinine (CREA). (B) H&E-stained sections of major organs including the heart, liver, spleen, lungs and kidneys. (C) Protein levels of inflammatory cytokines in the serum. Data are shown as mean ± S.D. (n = 3).

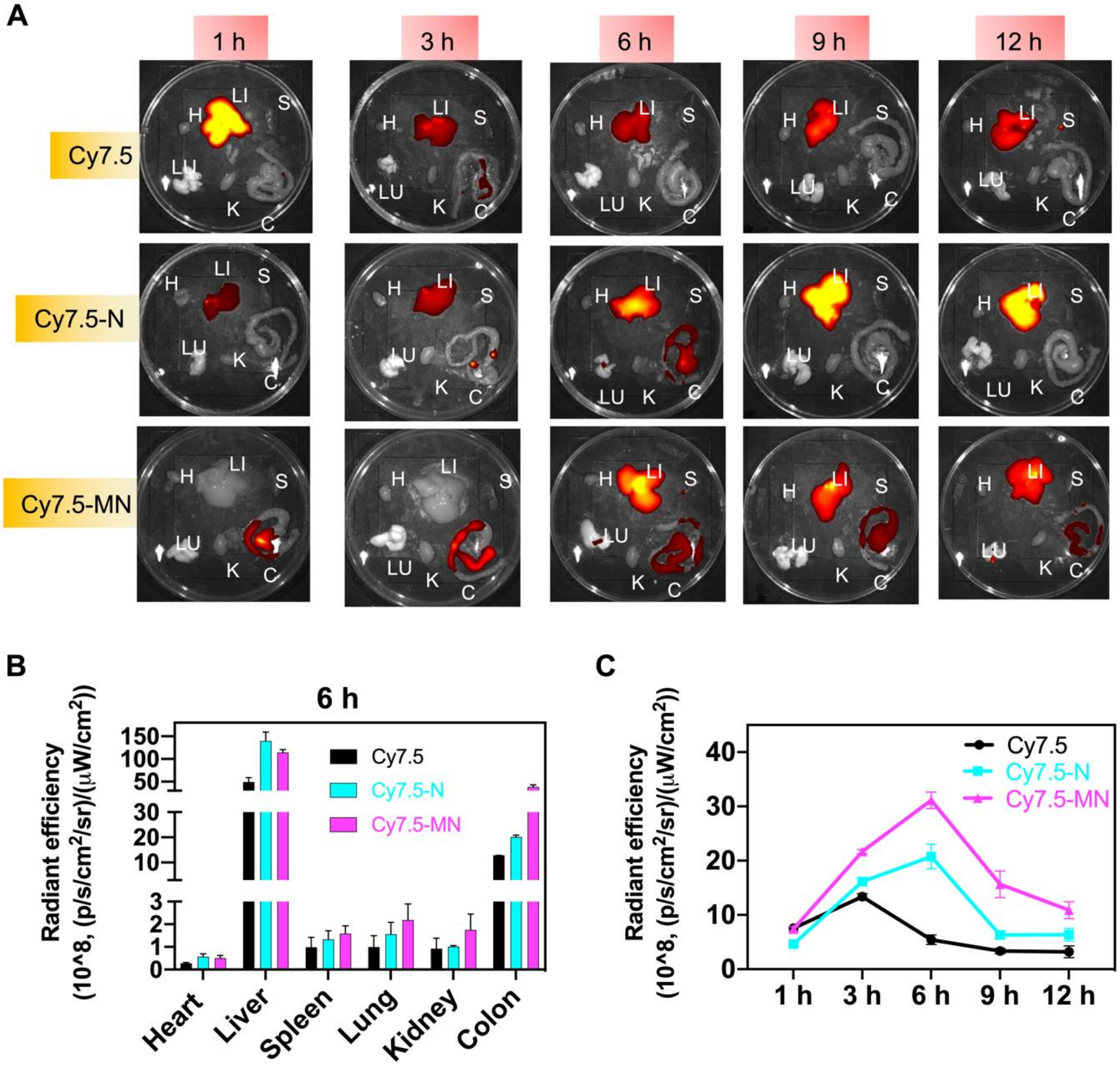

Figure S9. Biodistribution of free Cy7.5, Cy7.5-N and Cy7.5-MN. (A) Biodistribution of Cy7.5, Cy7.5-N and Cy7.5-MN in major organs and the colon at 1, 3, 6, 9, and 12 h post administration, visualized under *ex vivo* bioimaging. H, LI, S, LU, K, C represent the heart, liver, spleen, lungs, kidneys and colon, respectively. (B) Radiant efficiency of Cy7.5 in the major organs and colon at 6 h after administration. (C) Radiant efficiency of Cy7.5 in the colon determined at different time points post administration. Data are shown as mean ± S.D. (n = 3).

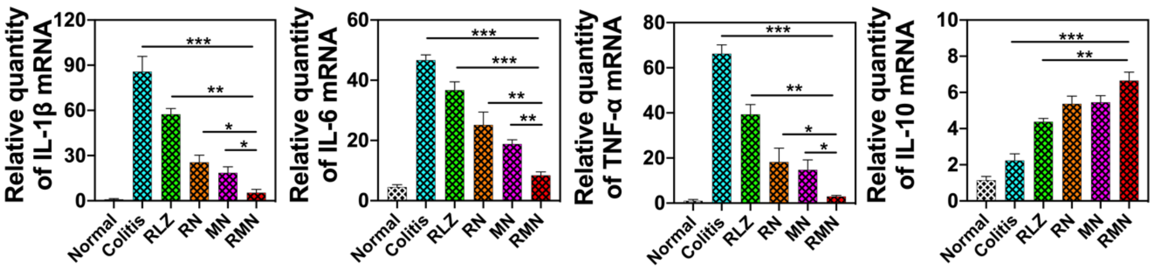

Figure S10. Relative mRNA levels of inflammatory cytokines from the colons *in vivo*. The mRNA levels of inflammatory cytokines were determined by qPCR. Data are shown as mean ± S.D. (n = 6). *****$p < 0.05$, ******$p < 0.01$, *******$p < 0.001$.

Table. S1 Body weights and DAI of mice on Day 7.

Body weight (% of body weight on Day 0)

Normal						Saline						RLZ					
103.48	104.49	102.46	102.40	104.45	104.29	77.04	78.05	75.38	75.00	84.62	75.36	80.85	84.06	83.92	73.72	89.38	87.50
RN						MN						RMN					
92.02	79.06	96.70	87.88	87.82	79.31	92.79	93.18	93.03	94.67	94.12	96.20	97.78	100.57	97.60	101.26	103.30	101.18

DAI

Normal						Saline						RLZ					
0	0	0	0	0	0	10	8	8	8	7	10	8	8	8	6	7	8
RN						MN						RMN					
4	8	4	6	6	8	5	4	4	6	4	4	5	3	2	0	0	0
